# Dexmedetomidine-Induced Contraction Involves CPI-17 Phosphorylation in Isolated Rat Aortas

**DOI:** 10.3390/ijms17101663

**Published:** 2016-09-30

**Authors:** Seong-Ho Ok, Seong-Chun Kwon, Jiseok Baik, Jeong-Min Hong, Jiah Oh, Jeong Yeol Han, Ju-Tae Sohn

**Affiliations:** 1 Department of Anesthesiology and Pain Medicine, Gyeongsang National University School of Medicine and Gyeongsang National University Hospital, Jinju-si 52727, Korea; mdoksh@naver.com; 2 Department of Physiology, Institute for Clinical and Translational Research, Catholic Kwandong University College of Medicine, Gangneung 25601, Korea; skwon2028@cku.ac.kr; 3 Department of Anesthesiology and Pain Medicine, Pusan National University Hospital, Biomed Research Institute, Pusan National University School of Medicine, Busan 49241, Korea; jidal75@naver.com (J.B.); ccarrot@daum.net (J.-M.H.); 4 Department of Anesthesiology and Pain Medicine, Gyeongsang National University Hospital, Jinju-si 52727, Korea; wldk0430@gmail.com; 5 Department of Anesthesiology and Pain Medicine, Gyeongsang National University Changwon Hospital, Changwon 51472, Korea; taszzang@hanmail.net; 6 Institute of Health Sciences, Gyeongsang National University, Jinju 52727, Korea

**Keywords:** dexmedetomidine, Rho-kinase, protein kinase C, calcium sensitization, phosphorylation-dependent inhibitory protein of myosin phosphatase, aorta

## Abstract

Dexmedetomidine, a highly selective α-2 adrenoceptor agonist, produces vasoconstriction, which leads to transiently increased blood pressure. The goal of this study was to investigate specific protein kinases and the associated cellular signal pathways responsible for the increased calcium sensitization induced by dexmedetomidine in isolated rat aortas, with a particular focus on phosphorylation-dependent inhibitory protein of myosin phosphatase (CPI-17). The effect of Y-27632 and chelerythrine on the dexmedetomidine-induced intracellular calcium concentration ([Ca^2+^]_i_) and tension were assessed using fura-2-loaded aortic strips. The effects of rauwolscine, Y-27632, chelerythrine, and ML-7 hydrochloride on the dexmedetomidine-induced phosphorylation of CPI-17 or of the 20-kDa regulatory light chain of myosin (MLC_20_) were investigated in rat aortic vascular smooth muscle cells. The effects of rauwolscine, Y-27632, and chelerythrine on the membrane translocation of Rho-kinase and protein kinase C (PKC) phosphorylation induced by dexmedetomidine were assessed. Y-27632 and chelerythrine each reduced the slopes of the [Ca^2+^]_i_-tension curves of dexmedetomidine-induced contraction, and Y-27632 more strongly reduced these slopes than did chelerythrine. Rauwolscine, Y-27632, chelerythrine, and ML-7 hydrochloride attenuated the dexmedetomidine-induced phosphorylation of CPI-17 and MLC_20_. Taken together, these results suggest that dexmedetomidine-induced contraction involves calcium sensitization, which appears to be mediated by CPI-17 phosphorylation via Rho-kinase or PKC.

## 1. Introduction

As a highly selective α-2 adrenoceptor agonist, dexmedetomidine induces sedation, reduces the opioid analgesic requirement, and attenuates the hemodynamic response to anesthesia and surgery [1,2]. Because of these characteristics, dexmedetomidine is widely used during the perioperative period [1,2]. Intravenous administration of dexmedetomidine produces a biphasic blood pressure response, which consists of transient hypertension followed by decreased blood pressure [3]. This initial transient hypertension is due to α-2 adrenoceptor stimulation of vascular smooth muscle, whereas the observed decrease in blood pressure is associated with a central sympatholytic effect evoked by dexmedetomidine [4,5,6,7,8]. In particular, a high dose or high concentration of dexmedetomidine produces severe hypertension, which appears to be associated with α-2 adrenoceptor stimulation of vascular smooth muscle [9,10,11,12].

Contraction of vascular smooth muscle is regulated by both calcium-dependent and calcium-sensitization mechanisms [13]. In the calcium-sensitization mechanism, the slope of the intracellular calcium concentration ([Ca^2+^]_i_)-tension curve induced by an agonist or drug is higher than that induced by the depolarization evoked by high KCl concentrations [13,14]. The underlying mechanism responsible for calcium sensitization is associated with the inhibition of myosin light chain phosphatase (MLCP), leading to increased phosphorylation of the 20-kDa regulatory light chain of myosin (MLC_20_) and subsequently enhanced contraction [13,14]. The inhibition of MLCP in vascular smooth muscle is mediated by phosphorylation of either the phosphorylation-dependent inhibitory protein of myosin phosphatase (CPI-17) or the myosin phosphatase target subunit of MLCP via either Rho-kinase or protein kinase C (PKC), which leads to attenuated dephosphorylation of MLC_20_ [13,14]. We previously reported that the slopes of [Ca^2+^]_i_-tension curves induced by dexmedetomidine are higher than those induced by high KCl in isolated rat aortas, suggesting that dexmedetomidine-induced contraction produces a relatively greater increase in calcium sensitization [15]. However, the specific protein kinases and associated cellular pathways primarily responsible for increased calcium sensitization in response to dexmedetomidine remain unknown. Therefore, the goal of this in vitro study was to investigate the specific protein kinase and associated cellular signaling pathways that contribute to the increased calcium sensitization induced by dexmedetomidine, with a particular focus on CPI-17 phosphorylation.

## 2. Results

**Figure 1:**
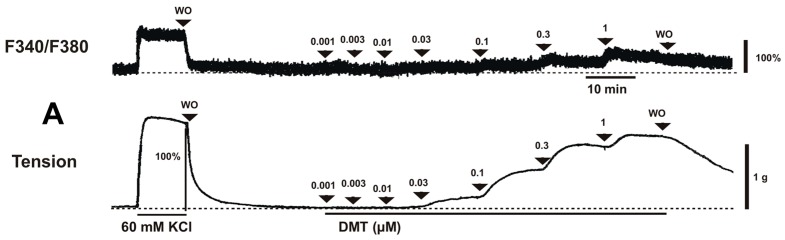

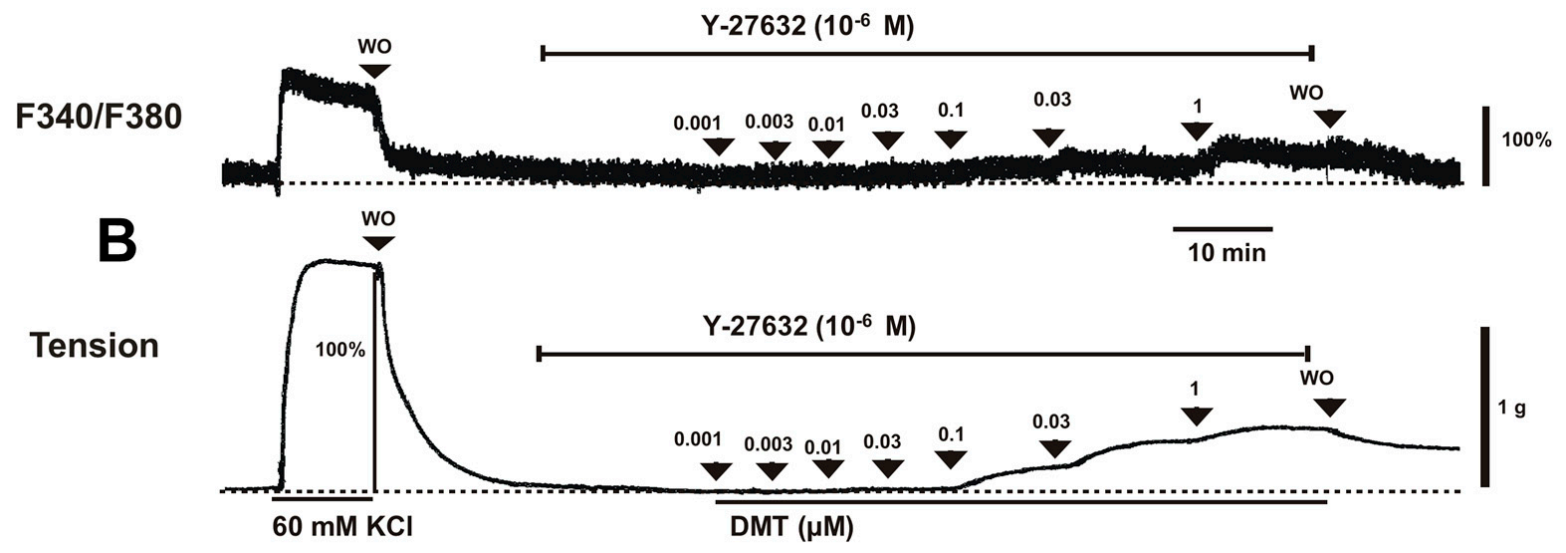
Effects of 60 mM KCl and dexmedetomidine (DMT) on the intracellular calcium concentration ([Ca^2+^]_i_) (upper trace) and muscle tension (lower trace) in the absence (**A**) and presence (**B**) of 10^−6^ M Y-27632 in endothelium-denuded rat aortas. The [Ca^2+^]_i_ of fura-2-loaded aortic strips was detected using a fluorometer and expressed as the F340/F380 ratio. After the effects of 60 mM KCl were determined, DMT (10^−9^ to 10^−6^ M) was cumulatively added. A value of 100% represents the 60 mM KCl-induced contraction or [Ca^2+^]_i_ increase measured before washout (WO) with Krebs solution.

**Figure 2:**
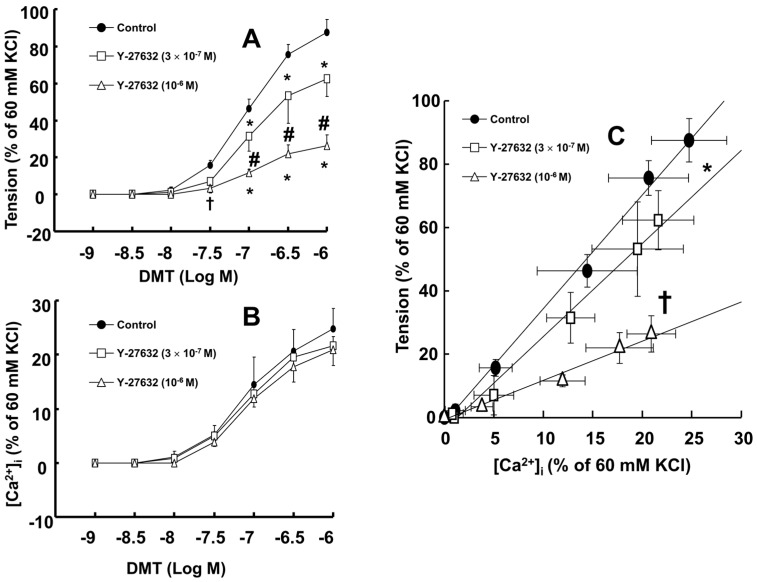
Effects of the cumulative addition of dexmedetomidine (DMT, 10^−9^ to 10^−6^ M) on muscle tension (**A**) and intracellular calcium concentrations ([Ca^2+^]_i_) (**B**) in the absence or presence of 3 × 10^−7^ M or 10^−6^ M Y-27632 in endothelium-denuded rat aortas. A value of 100% represents the 60 mM KCl-induced [Ca^2+^]_i_ increase and muscle tension measured before washout with Krebs solution; (**C**) The [Ca^2+^]_i_-tension relationship was constructed by measurements of the cumulative addition of DMT (10^−9^ to 10^−6^ M) in the absence or presence of Y-27632. Each point represents the mean of five experiments, and the standard deviation (SD) is shown by both the vertical and horizontal bars. The effects of Y-27632 on the DMT concentration-response curves were analyzed using two-way repeated measures analysis of variance (ANOVA) followed by Bonferroni’s post hoc test. The slopes of the [Ca^2+^]_i_-tension curves induced by DMT were calculated using linear regression, and the effect of Y-27632 on the slope was analyzed using one-way ANOVA followed by Bonferroni’s post hoc test. (**A**,**B**): * *p* < 0.001 and ^†^ *p* < 0.01 versus control. ^#^ *p* < 0.001 versus 3 × 10^−7^ M Y-27632; (**C**): slope; * *p* < 0.05 and ^†^ *p* < 0.001 versus control.

**Figure 3:**
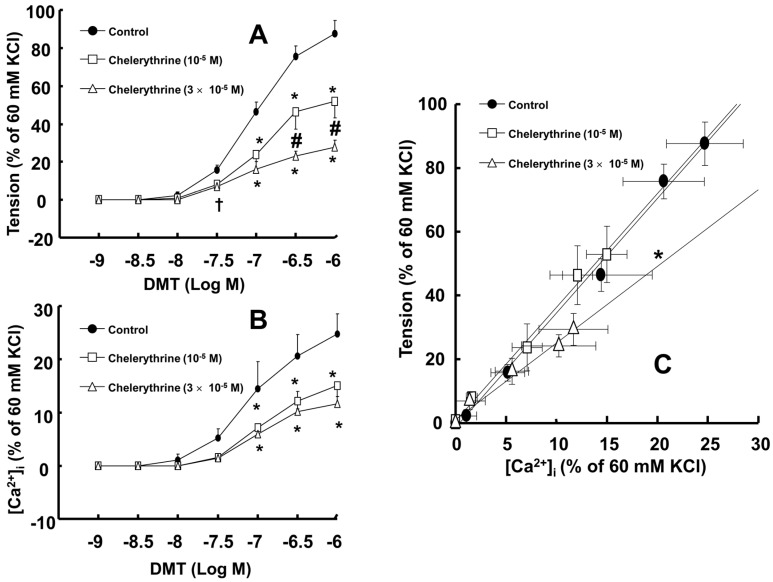
Effects of the cumulative addition of dexmedetomidine (DMT, 10^−9^ to 10^−6^ M) on muscle tension (**A**) and intracellular calcium concentrations ([Ca^2+^]_i_) (**B**) in the absence or presence of 10^−5^ M or 3 × 10^−5^ M chelerythrine in endothelium-denuded rat aortas. A value of 100% represents the 60 mM KCl-induced [Ca^2+^]_i_ increase and muscle tension measured before washout with Krebs solution; (**C**) The [Ca^2+^]_i_-tension relationship was constructed by the cumulative addition of DMT (10^−9^ to 10^−6^ M) in the absence or presence of chelerythrine. Each point represents the mean of five experiments, and the SD is shown by both the vertical and horizontal bars. The effects of chelerythrine on the DMT concentration-response curves were analyzed using two-way repeated measures analysis of variance (ANOVA) followed by Bonferroni’s post hoc test. The slopes of the [Ca^2+^]_i_-tension curves induced by DMT were calculated using linear regression, and the effect of chelerythrine on the slope was analyzed using one-way ANOVA followed by Bonferroni’s post hoc test. (**A**,**B**): * *p* < 0.001 and ^†^ *p* < 0.05 versus control. ^#^ *p* < 0.001 versus 10^−5^ M chelerythrine; (**C**): slope; * *p* < 0.01 versus control.

Dexmedetomidine (3 × 10^−8^ to 10^−6^ M) induced vasoconstriction (*p* < 0.001 versus control; Figure 1A and Figure 2A) and increased [Ca^2+^]_i_ (*p* < 0.01 versus control; Figure 1A and Figure 2B). Y-27632 (3 × 10^−7^ and 10^−6^ M) attenuated the dexmedetomidine-induced contraction in a concentration-dependent manner (*p* < 0.001 versus control at 10^−7^ to 10^−6^ M; Figure 1A,B and Figure 2A) without changing [Ca^2+^]_i_ (Figure 1A,B and Figure 2B). Thus, Y-27632 (3 × 10^−7^ and 10^−6^ M) shifted the slope of the [Ca^2+^]_i_-tension curves to the lower-right compared with that of the control, suggesting that Y-27632 decreases Ca^2+^ sensitization (*p* < 0.05; Figure 2C). Chelerythrine (10^−5^ and 3 × 10^−5^ M) attenuated dexmedetomidine-induced contraction in a concentration-dependent manner (*p* < 0.001 versus control at 10^−7^ to 10^−6^ M; Figure 3A) and inhibited dexmedetomidine-induced [Ca^2+^]_i_ increases (*p* < 0.001 versus control at 10^−7^ to 10^−6^ M; Figure 3B). Only a high concentration of chelerythrine (3 × 10^−5^ M) reduced the slopes of the [Ca^2+^]_i_-tension curves induced by dexmedetomidine compared with those of the control (*p* < 0.01; Figure 3C).

**Figure 4:**
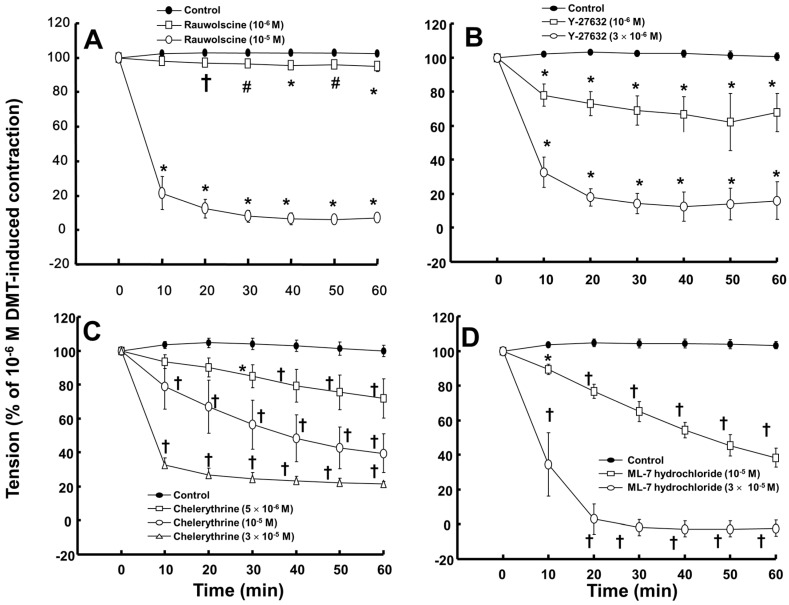
Effect of rauwolscine ((**A**), *n* = 6), Y-27632 ((**B**), *n* = 6), chelerythrine ((**C**), *n* = 6), and ML-7 hydrochloride ((**D**), *n* = 6) on the dexmedetomidine (DMT)-induced contraction in endothelium-denuded aortas. All data are expressed as the mean ± SD and the percentage of the maximal contraction induced by DMT (10^−6^ M). N indicates the number of rats from which descending thoracic aortic rings were derived or the number of isolated rat aortic rings. The effects of each inhibitor (rauwolscine, Y-27632, chelerythrine, and ML-7 hydrochloride) on the DMT concentration-response curves were analyzed using two-way repeated measures analysis of variance followed by Bonferroni’s post hoc test. (**A**,**B**): ^†^ *p* < 0.05, ^#^ *p* < 0.01, and * *p* < 0.001 versus control; (**C**,**D**): * *p* < 0.01 and ^†^ *p* < 0.001 versus control.

Rauwolscine (10^−6^ and 10^−5^ M) and ML-7 hydrochloride (10^−5^ and 3 × 10^−5^ M) attenuated the contraction induced by dexmedetomidine (10^−6^ M) (Figure 4A,D; rauwolscine: *p* < 0.05 versus control; ML-7 hydrochloride: *p* < 0.01 versus control). The highest concentrations of rauwolscine (10^−5^ M) and ML-hydrochloride (3 × 10^−5^ M) nearly abolished the dexmedetomidine (10^−6^ M)-induced contraction (Figure 4A,D; *p* < 0.001 versus control at 20 to 60 min). Y-27632 (10^−6^ and 3 × 10^−6^ M) also attenuated the dexmedetomidine (10^−6^ M)-induced contraction (Figure 4B; *p* < 0.001 versus control). The highest concentration of Y-27632 (3 × 10^−6^ M) nearly abolished the dexmedetomidine (10^−6^ M)-induced contraction (Figure 4B; *p* < 0.001 versus control at 10 to 60 min), and chelerythrine (5 × 10^−6^ to 3 × 10^−5^ M) attenuated the dexmedetomidine (10^−6^ M)-induced contraction (Figure 4C; *p* < 0.01 versus control at 30 to 60 min). In addition, the highest concentration of chelerythrine (3 × 10^−5^ M) nearly abolished the dexmedetomidine (10^−6^ M)-induced contraction (Figure 4C; *p* < 0.001 versus control at 10 to 60 min).

**Figure 5:**
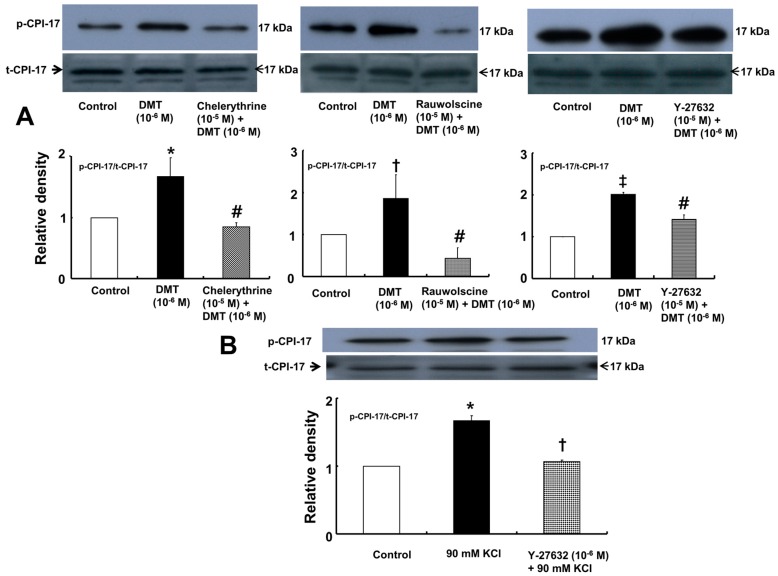
(**A**) Effect of chelerythrine (*n* = 4), rauwolscine (*n* = 4), and Y-27632 (*n* = 4) on the phosphorylation of phosphorylation-dependent inhibitory protein of myosin phosphatase (CPI-17) at Thr38 induced by dexmedetomidine (DMT, 10^−6^ M) in rat aortic vascular smooth muscle cells (VSMCs). VSMCs were treated with 10^−6^ M DMT alone for 4 min or 10^−6^ M DMT for 4 min after pretreatment with 10^−5^ M chelerythrine, 10^−5^ M rauwolscine, or 10^−5^ M Y-27632 for 1 h. The data are presented as the mean ± SD. N indicates the number of independent experiments. p-CPI-17: phosphorylated CPI-17, t-CPI-17: total CPI-17. Effects of chelerythrine, rauwolscine, and Y-27632 on the CPI-17 phosphorylation induced by DMT were analyzed using one-way analysis of variance followed by Bonferroni’s post hoc test. * *p* < 0.01, ^†^ *p* < 0.05 and ^‡^ *p* < 0.001 versus control. ^#^ *p* < 0.001 versus 10^−6^ M DMT alone; (**B**) Effect of Y-27632 (*n* = 3) on 90 mM KCl-induced phosphorylation of CPI-17 at Thr38 in rat aortic VSMCs. VSMCs were treated with 90 mM KCl alone for 10 min or with 90 mM KCl for 10 min after pretreatment with 10^−5^ M Y-27632 for 1 h. The data are presented as the mean ± SD. N indicates the number of independent experiments. The effects of Y-27632 on the CPI-17 phosphorylation induced by 90 mM KCl were analyzed using one-way analysis of variance followed by Bonferroni’s post hoc test. * *p* < 0.001 versus control. ^†^ *p* < 0.001 versus 90 mM KCl alone.

**Figure 6:**
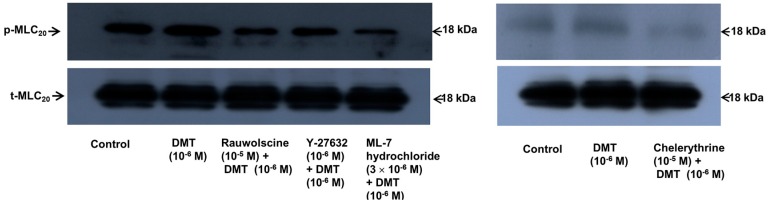

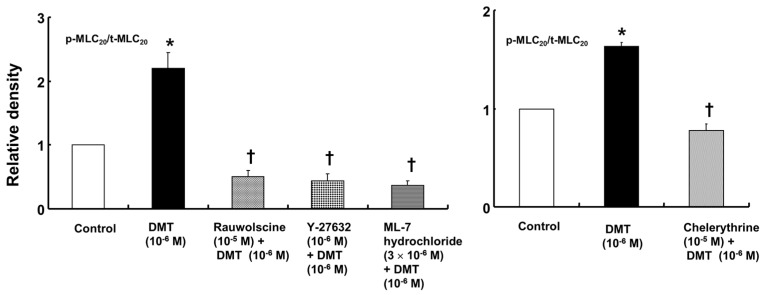
Effect of rauwolscine (*n* = 4), Y-27632 (*n* = 4), ML-7 hydrochloride (*n* = 4), and chelerythrine (*n* = 4) on the phosphorylation of 20-kDa regulatory light chain of myosin (MLC_20_) at Ser19 induced by dexmedetomidine (DMT, 10^−6^ M) in rat aortic vascular smooth muscle cells (VSMCs). VSMCs were treated with 10^−6^ M DMT alone for 6 min or 10^−6^ M DMT for 6 min after pretreatment with 10^−5^ M rauwolscine, 10^−6^ M Y-27632, 3 × 10^−6^ M ML-7 hydrochloride, or 10^−5^ M chelerythrine for 1 h. The data are presented as the mean ± SD. N indicates the number of independent experiments. The effects of rauwolscine, Y-27632, ML-7 hydrochloride, and chelerythrine on the phosphorylation of MLC_20_ at Ser19 induced by DMT were analyzed using one-way analysis of variance followed by Bonferroni’s post hoc test. * *p* < 0.001 versus control. ^†^ *p* < 0.001 versus 10^-6^ M DMT alone. p-MLC_20_: phosphorylated MLC_20_, t-MLC_20_: total MLC_20_.

**Figure 7:**
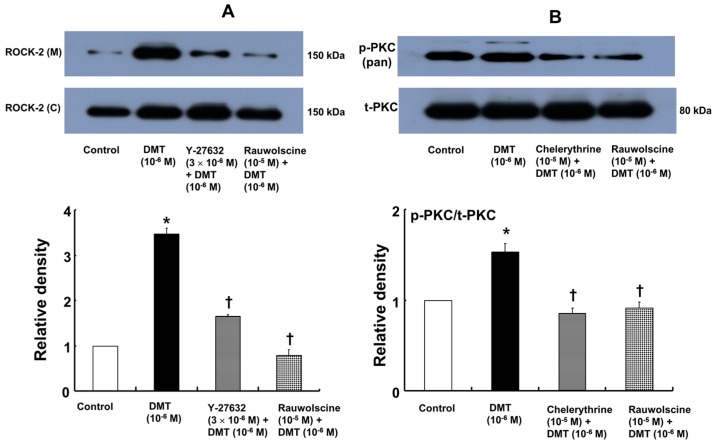
(**A**) Effect of Y-27632 (*n* = 3) and rauwolscine (*n* = 3) on the membrane translocation of Rho-kinase (ROCK-2) induced by dexmedetomidine (DMT, 10^−6^ M) in rat aortic vascular smooth muscle cells (VSMCs). VSMCs were treated with 10^−6^ M DMT alone for 10 min or with 10^−6^ M DMT for 10 min after pretreatment with 3 × 10^−6^ M Y-27632 or 10^−5^ M rauwolscine for 1 h. The translocation of ROCK-2 from the cytosol (C) to the membrane (M) was examined by western blot as described in the methods. The data are presented as the mean ± SD. N indicates the number of independent experiments. The effects of Y-27632 and rauwolscine on the membrane translocation of ROCK-2 induced by DMT were analyzed using one-way analysis of variance followed by Bonferroni’s post hoc test. * *p* < 0.001 versus control. ^†^ *p* < 0.001 versus 10^−6^ M DMT alone; (**B**) Effect of chelerythrine (*n* = 4) and rauwolscine (*n* = 4) on the protein kinase C (PKC) pan-phosphorylation induced by DMT (10^−6^ M) in rat aortic VSMCs. VSMCs were treated with 10^−6^ M DMT alone for 4 min or 10^−6^ M DMT for 4 min after pretreatment with 10^−5^ M chelerythrine or 10^−5^ M rauwolscine for 1 h. The data are presented as the mean ± SD. N indicates the number of independent experiments. The effects of chelerythrine and rauwolscine on the PKC pan-phosphorylation induced by DMT were analyzed using one-way analysis of variance followed by Bonferroni’s post hoc test. * *p* < 0.001 versus control. ^†^ *p* < 0.001 versus 10^−6^ M DMT alone. p-PKC (pan): phosphorylated PKC (pan), t-PKC: total PKC.

Dexmedetomidine (10^−6^ M) induced CPI-17 phosphorylation at Thr38 in rat aortic vascular smooth muscle cells (VSMCs) (Figure 5A; *p* < 0.05), whereas pretreatment with chelerythrine (10^−5^ M) or rauwolscine (10^−5^ M) attenuated this effect (Figure 5A; *p* < 0.001 versus 10^−6^ M dexmedetomidine alone). In addition, Y-27632 (10^−5^ M) also attenuated the dexmedetomidine-induced CPI-17 phosphorylation (Figure 5A; *p* < 0.001 versus 10^−6^ M dexmedetomidine alone). In addition, 90 mM KCl induced CPI-17 phosphorylation in rat aortic VSMCs (Figure 5B; *p* < 0.001 versus control), whereas Y-27632 attenuated the 90 mM KCl-induced CPI-17 phosphorylation (Figure 5B; *p* < 0.001). Dexmedetomidine (10^−6^ M) induced the phosphorylation of MLC_20_ at Ser19 in rat aortic VSMCs (Figure 6; *p* < 0.001 versus control), whereas pretreatment with rauwolscine (10^−5^ M), Y-27632 (10^−6^ M), ML-7 hydrochloride (3 × 10^−6^ M) and chelerythrine (10^−5^ M) attenuated this effect (Figure 6; *p* < 0.001 versus 10^−6^ M dexmedetomidine alone). Dexmedetomidine (10^−6^ M) also induced membrane translocation of Rho-kinase (Figure 7A; *p* < 0.001 versus control), and this effect was inhibited by Y-27632 (3 × 10^−6^ M) and rauwolscine (10^−5^ M) (Figure 7A; *p* < 0.001 versus 10^−6^ M dexmedetomidine alone). Furthermore, dexmedetomidine (10^−6^ M) induced PKC phosphorylation (Figure 7B; *p* < 0.001), which was inhibited by chelerythrine (10^−5^ M) and rauwolscine (10^−5^ M) (Figure 7B; *p* < 0.001 versus 10^−6^ M dexmedetomidine alone).

## 3. Discussion

This is first study to suggest that dexmedetomidine-induced contraction involves the calcium sensitization mediated by CPI-17 phosphorylation. The major findings of this in vitro study are as follows: (1) the magnitudes of the reduction of the slopes of [Ca^2+^]_i_-tension curves induced by dexmedetomidine were greater in aortas pretreated with Y-27632 than in those pretreated with chelerythrine; (2) rauwolscine, Y-27632 and chelerythrine attenuated the dexmedetomidine-induced CPI-17 phosphorylation; and (3) rauwolscine, Y-27632, chelerythrine, and ML-7 hydrochloride attenuated the dexmedetomidine-induced MLC_20_ phosphorylation.

A previous study demonstrated that the slope of the [Ca^2+^]_i_-tension curve induced by dexmedetomidine is steeper than the slope of the [Ca^2+^]_i_-tension curve induced by high KCl, suggesting that dexmedetomidine-induced α-2 adrenoceptor-mediated contraction would be mediated by calcium sensitization [15]. However, the downstream cellular signal pathways associated with specific protein kinases that contribute to calcium sensitization remain unknown. KCl-induced contraction involves both the activation of myosin light chain kinase and the calcium-calmodulin-stimulated enhancement of Rho-kinase translocation that contributes to calcium sensitization via an increase in [Ca^2+^]_i_ [16,17]. However, a contractile agonist produces contraction due to greater calcium sensitization involving Rho-kinase and PKC [14,18]. In terms of downstream effectors that contribute to the calcium sensitization induced by Rho-kinase and the PKC activation by an agonist, PKC induces the phosphorylation of CPI-17, whereas Rho-kinase induces the phosphorylation of both CPI-17 and the myosin phosphatase target subunit of MLCP [14,19]. Therefore, the Y-27632 induced greater reduction of the slopes of the [Ca^2+^]_i_-tension curves induced by dexmedetomidine compared with those induced by chelerythrine (Figure 2C and Figure 3C; slope: 10^−6^ M Y-27632 = 1.27 ± 0.39 versus 3 × 10^−5^ M chelerythrine = 2.37 ± 0.53; *p* < 0.01); this may be ascribed to an additional inhibitory effect of Y-27632 on the dexmedetomidine-induced phosphorylation of the myosin phosphatase target subunit, leading to enhanced calcium sensitization. Thus, further studies regarding the effect of dexmedetomidine on the phosphorylation of the myosin phosphatase target subunit are needed to elucidate the detailed mechanism. Because cytoskeletal organization, including stress fiber and focal adhesion formation, is controlled by Rho-kinase-induced myosin light chain phosphorylation, the dexmedetomidine-induced increase in the slope of the [Ca^2+^]_i_-tension curves may be associated with cytoskeletal rearrangement [19,20].

In agreement with previous reports, rauwolscine attenuated dexmedetomidine-induced contraction, suggesting that dexmedetomidine-induced contraction involves activation of the α-2 adrenoceptor [15,21,22,23]. Similar to previous reports, chelerythrine and Y-27632 attenuated dexmedetomidine-induced contraction in a concentration-dependent manner, suggesting that dexmedetomidine-induced contraction is mediated by PKC and Rho-kinase [15,21,23,24]. Consistent with a previous report, ML-7 hydrochloride attenuated dexmedetomidine-induced contraction, suggesting that dexmedetomidine-induced contraction involves activation of myosin light chain kinase [24]. In the current study, dexmedetomidine-mediated contraction involved myosin light chain kinase activation; thus, the observed increase in dexmedetomidine-induced [Ca^2+^]_i_ suggests a contribution to myosin light chain kinase activation through the calcium-calmodulin complex [13,23]. Taken together, these results suggest that dexmedetomidine-induced contraction is mediated by pathways involving α-2 adrenoceptor, PKC, Rho-kinase, and myosin light chain kinase. However, although the concentrations of these inhibitors were selected based on previous studies, non-specific action of these inhibitors may affect dexmedetomidine-induced contraction [25,26,27,28].

The calcium sensitization contributing to smooth muscle contraction is mediated by the inhibition of MLCP [13]. The binding of an agonist acting on G-protein-coupled receptors induces the hydrolysis of phosphatidylinositol 4,5-bisphosphate by phospholipase C and the hydrolysis of phosphatidylcholine by phospholipase D, leading to the production of diacylglycerol which activates PKC, which subsequently phosphorylates CPI-17 and causes enhanced contraction via inhibition of MLCP [13,14,18]. In addition, agonist-receptor binding stimulates Rho-kinase via RhoA-GTP and subsequently phosphorylates the myosin phosphatase target subunit of MLCP, leading to enhanced contraction via the inhibition of MLCP [14]. In addition, agonist-induced Rho-kinase activation causing smooth muscle contraction has been reported to phosphorylate CPI-17 as a downstream effector, leading to enhanced contraction via the inhibition of MLCP [18,19,29,30]. Similar to previous reports, Y-27632 or chelerythrine attenuated the dexmedetomidine-induced phosphorylation of CPI-17 at Thr38, suggesting that CPI-17 involved in dexmedetomidine-induced contraction is a downstream effector activated by Rho-kinase or PKC [18,19,29,30]. Consistent with the isometric tension measurements in the current study, rauwolscine, Y-27632, chelerythrine, and ML-7 hydrochloride attenuated the dexmedetomidine-induced phosphorylation of MLC_20_. The relative attenuation of dexmedetomidine-induced CPI-17 phosphorylation by Y-27632 appears to be lower than that induced by chelerythrine (Figure 5), whereas the relative inhibition of dexmedetomidine-induced MLC_20_ phosphorylation by Y-27632 appears to be higher than that induced by chelerythrine (Figure 6). Although we did not examine the effect of Y-27632 on the dexmedetomidine-induced phosphorylation of the myosin phosphatase target subunit in the current study, Y-27632 induced a greater attenuation of the slopes of the [Ca^2+^]_i_-tension curves than that induced by chelerythrine (Figure 2C and Figure 3C); thus, we suppose that the enhanced inhibitory effect of Y-27632 on dexmedetomidine-induced MLC_20_ phosphorylation may be due to the additional inhibition of the phosphorylation of the myosin phosphatase target subunit induced by Rho-kinase activation by dexmedetomidine. Similar to a previous report, Y-27632 attenuated the CPI-17 phosphorylation induced by high levels of KCl, suggesting that the 90 mM KCl-induced contraction is mediated partially by Rho-kinase-induced CPI-17 phosphorylation [17]. In addition, in the current study, Y-27632 and rauwolscine attenuated the dexmedetomidine-induced membrane translocation of Rho-kinase, and both chelerythrine and rauwolscine attenuated the dexmedetomidine-induced PKC phosphorylation. Taken together, these results suggest that the dexmedetomidine-induced phosphorylation of MLC_20_ is mediated by pathways involving α-2 adrenoceptor, Rho-kinase, PKC, and myosin light chain kinase. Further study regarding the upstream signal pathways associated with Rho-kinase and PKC that contribute to dexmedetomidine-induced CPI-17 phosphorylation is needed.

An initial loading dose or bolus administration of dexmedetomidine produces transient hypertension and increased systemic vascular resistance [31,32]. In addition, high doses or high concentrations of dexmedetomidine cause hypertension in humans [9,10,11,12]. Although our results help to explain these observations, some limitations of our study should be considered. First, dexmedetomidine-induced α-2 adrenoceptor-mediated endothelial nitric oxide release is known to attenuate the corresponding dexmedetomidine-induced vasoconstriction, but we used endothelium-denuded aortas in the current study [33,34]. Second, the total peripheral vascular resistance is primarily determined by small arterioles, although we used rat aortas, which are regarded as conduit vessels, in the current study [35]. Although we used cultured rat aortic VSMCs between passages two and ten, because prolonged culture of vascular myocytes may grossly change their phenotype and biochemical properties, the use of cultured rat aortic VSMCs instead of fresh tissue in the current study may affect the Western blot results. Even with these limitations, the vasoconstriction evoked by dexmedetomidine-induced CPI-17 phosphorylation-mediated calcium sensitization may contribute to the increased blood pressure or systemic vascular resistance observed in previous studies involving humans [9,10,11,12].

## 4. Materials and Methods

The Institutional Animal Care and Use Committee at Gyeongsang National University and at Catholic Kwandong University approved all the experimental procedures and protocols (GLA-130627-R0041, 17 June 2013), and all experimental procedures were performed in accordance with the Guide for the Care and Use of Laboratory Animals prepared by the Institute for Laboratory Animal Research.

### 4.1. Fura-2 Loading and the Simultaneous Measurements of the Intracellular Calcium Concentration ([Ca^2+^]_i_) and Tension

Fura-2 loading and simultaneous measurements of [Ca^2+^]_i_ and tension were performed as described earlier [36]. Male Sprague-Dawley rats weighing 250–300 g were sacrificed by intraperitoneal administration of pentobarbital sodium (50 mg/mL) followed by exsanguination. The descending thoracic aorta was isolated and dissected free from the surrounding connective tissue and fat, removed under microscopic guidance, and placed in Krebs solution (118 mM NaCl, 4.7 mM KCl, 1.2 mM MgSO_4_, 1.2 mM KH_2_PO_4_, 2.5 mM CaCl_2_, 25 mM NaHCO_3_, and 11 mM glucose). A total of 20 rats were used in this experiment. [Ca^2+^]_i_ was measured using the fluorescent Ca^2+^ indicator fura-2. Vascular smooth muscle strips were preincubated with the acetoxymethyl ester of fura-2 (fura-2/AM, 10 µM) in the presence of 0.02% Cremophor EL for 5–6 h at room temperature. Fura-2-loaded tissues were then transferred to a temperature-controlled 7 mL organ bath in a fluorimeter (CAF-100; Jasco, Tokyo, Japan) and washed with Krebs solution at 37 °C for 20 min to remove uncleaved fura-2/AM. Isometric contraction of the muscle was recorded using a force-displacement transducer (MLT050, AD Instruments, Colorado Springs, CO, USA). The muscle strips were alternately illuminated (48 Hz) at excitation wavelengths of 340 and 380 nm. The light emitted from the tissue (F340 and F380) was measured by a fluorometer through a 500-nm filter, and the ratio of F340/F380 was used as a measure of [Ca^2+^]_i_. The absolute [Ca^2+^]_i_ was not calculated in this experiment because the dissociation constant of the fluorescence indicator for Ca^2+^ in cytosol may differ from that measured in vitro [37]. Therefore, the F340/F380 ratios obtained in resting and 60 mM KCl-stimulated muscle were expressed as 0% and 100%, respectively. Additionally, the maximal tension induced by 60 mM KCl was taken as 100%. Muscle tensions and F340/F380 ratios were recorded by PowerLab/400 using the chart program (AD Instruments). Muscle strips were placed under an initial 3.0 g resting tension. All the strips obtained from the same animal were used in different experimental protocols. The simultaneously measured [Ca^2+^]_i_-tension relationships obtained by cumulative addition of dexmedetomidine (10^−9^–10^−6^ M) were generated in the absence or presence of either a Rho-kinase inhibitor (Y-27632, 3 × 10^−7^ and 10^−6^ M) or a PKC inhibitor (chelerythrine, 10^−5^ and 3 × 10^−5^ M). Inhibitors were added to the organ bath 15 min before the cumulative application of dexmedetomidine and remained until the end of the measurement.

### 4.2. Preparation of Aortic Rings for Tension Measurement

Aortic rings were isolated for tension measurements as previously described [21,38]. Male Sprague-Dawley rats weighing 250–300 g were anesthetized by an intramuscular administration of Zoletil 50 (125 mg tiletamine–HCl + 125 mg zolazepam base/5 mL, 15 mg/kg; Virbac Laboratories, Carros, France). Zoletil 50 had no significant effect on the 60 mM KCl-induced contraction in the preliminary experiment. The descending thoracic aorta was removed and dissected free from its surrounding connective tissue and fat under microscopic guidance and placed in Krebs solution. The aorta was then cut into 2.5 mm rings and suspended on Grass isometric transducers (FT-03, Grass Instrument, Quincy, MA, USA) under a 3.0 g resting tension in a 10 mL Krebs bath at 37 °C. The aorta was continuously aerated with 95% O_2_ and 5% CO_2_ to maintain the pH value at 7.35–7.45. A 3.0 g resting tension was used to equilibrate the rings for 120 min, and the bath solution was changed every 30 min. A 25-gauge needle was inserted into the aortic lumen and the aortic rings were gently rubbed for a few seconds to remove the endothelium. After the stable maintenance of the contraction induced by phenylephrine (10^−8^ M), endothelial denudation was confirmed when <10% relaxation was observed in response to acetylcholine (10^−5^ M). After removing the Krebs solution containing phenylephrine from the organ bath to relax the isometric tension to baseline, the following experimental protocols were performed. A single ring was used to generate the concentration–response curve stimulated by dexmedetomidine. Since nitric oxide released by endothelial α-2 adrenoceptor inhibits dexmedetomidine-induced contraction, a nitric oxide synthase inhibitor (*N*^ω^-nitro-l-arginine methyl ester, 10^−4^ M) and a cyclooxygenase inhibitor (indomethacin, 10^−5^ M) were added in the Krebs solution for subsequent experimental protocols to inhibit the production of endogenous nitric oxide and endogenous prostacyclin, respectively, from any residual endothelium [22,33].

### 4.3. Experimental Protocol

The effects of the α-2 adrenoceptor inhibitor rauwolscine, the Rho-kinase inhibitor Y-27632, the PKC inhibitor chelerythrine, and the myosin light chain kinase inhibitor ML-7 hydrochloride on dexmedetomidine (10^−6^ M)-induced contraction were assessed by continuously measuring the dexmedetomidine (10^−6^ M)-induced contraction for 60 min. After dexmedetomidine (10^−6^ M) produced a sustained and stable contraction, rauwolscine (10^−6^ and 10^−5^ M), Y-27632 (10^−6^ and 3 × 10^−6^ M), chelerythrine (5 × 10^−6^, 10^−5^ and 3 × 10^−5^ M), and ML-7 hydrochloride (10^−5^ and 3 × 10^−5^ M) were added to an organ bath, and the isometric tension was continuously monitored for 60 min. The various concentrations of several inhibitors had no effect on the baseline resting tension in a preliminary experiment. In addition, we selected the various concentrations of several inhibitors (Y-27632, chelerythrine, rauwolscine, and ML-7 hydrochloride) based on the concentrations used in the previous studies [25,26,27,28].

### 4.4. Cell Culture

VSMCs were isolated from rat thoracic aortas using enzymatic dissociation and cultured in Dulbecco’s modified Eagle’s medium supplemented with 10% heat-inactivated fetal bovine serum, 2 mM l-glutamine, 100 U/mL penicillin, and 100 µg/mL streptomycin, as described earlier [36]. Cells were subcultured twice per week by harvesting with trypsin/ethylenediaminetetraacetic acid (EDTA) and seeding into flasks at a density of 7.5 × 10^5^/mm^2^. For current experiments, cells between passages two and ten were seeded into dishes (10^7^/100-mm dish), fed every other day, and used at confluence (6–7 days). Cells were serum deprived overnight prior to treatment. As dexmedetomidine (10^−6^ M) highly increased calcium sensitization and dexmedetomidine (10^−6^ M)-induced contraction was approximately equivalent in magnitude to that of the 60 mM KCl-induced contraction, we used 10^−6^ M dexmedetomidine to detect phosphorylation of various protein kinases induced by dexmedetomidine in the current experiment.

### 4.5. Western Blot Analysis

Western blot analysis was performed according to the method described by Ok et al. [36]. Membrane and cytosolic fractions were isolated from cells using a Mem-PER™ eukaryotic membrane protein extraction reagent kit (Thermo Scientific, Rockford, IL, USA) according to the manufacturer’s specifications. Protein concentrations were determined using the Bradford method [39]. Samples for gel loading were prepared by mixing equal volumes of 2× sodium dodecyl sulfate sample buffer (0.1 M Tris-HCl, 20% glycerol, 4% sodium dodecyl sulfate, and 0.01% bromophenol blue) and supernatant fractions from the lysates. Aliquots of 30 µg proteins were separated by 10% or 13.5% sodium dodecyl sulfate-polyacrylamide gel electrophoresis for 90 min at 110 V. The separated proteins were electrophoretically transferred to polyvinylidene difluoride membranes for 1 h at 190 mA. Then, the membranes were blocked in Tris-buffered saline (pH 7.0) containing 5% *w*/*v* nonfat dried milk for 2 h at room temperature and incubated overnight at 4 °C with specific primary antibodies (anti-PKC, anti-phospho-PKC [pan], anti-MLC_20_, anti-phospho-MLC_20_, anti-CPI-17, and anti-phospho-CPI-17) diluted 1:1000 in 5% *w*/*v* skim milk in Tris-buffered saline containing Tween-20 (TBST). After washing the membranes in TBST, bound antibodies were incubated with horseradish peroxidase-conjugated anti-goat, anti-rabbit, or anti-mouse IgG diluted 1:5000 in TBST containing 5% *w*/*v* skim milk for 1 h at room temperature. The membranes were washed in TBST, and the immunoreactive bands were detected by chemiluminescence (SuperSignal^®^ West Pico Chemiluminescent Substrate; Thermo Scientific, Rockford, IL, USA) using an X-ray film (*^Super^RX-N* Fuji Medical X-ray Film, Tokyo, Japan).

### 4.6. Materials

All drugs used in these experiments were the highest purity available commercially. L-NAME, indomethacin, acetylcholine, chelerythrine, rauwolscine, and ML-7 hydrochloride were obtained from Sigma-Aldrich (St. Louis, MO, USA). Y-27632 was obtained from Calbiochem (La Jolla, CA, USA). Anti-PKC, anti-phospho-PKC (Pan), anti-MLC_20_, and anti-phospho-MLC_20_ at Ser19 were obtained from Cell Signaling Technology (Beverly, MA, USA). Anti-CPI-17 and anti-phospho-CPI-17 at Thr38 were obtained from Santa Cruz Biotechnology (Santa Cruz, CA, USA). Fura-2/AM was obtained from Molecular Probes (Eugene, OR, USA). Dulbecco’s modified Eagle’s medium, fetal bovine serum, penicillin, streptomycin, trypsin/EDTA, and glutamine were supplied by Gibco BRL (Rockville, MD, USA). All concentrations are expressed as the final molar concentration in the organ bath. Indomethacin and ML-7 hydrochloride were dissolved in dimethyl sulfoxide (final organ bath concentration, <0.1%). Unless otherwise stated, all drugs were dissolved and diluted in distilled water.

### 4.7. Data Analysis

Values are expressed as the means ± SD. The vasodilation induced by rauwolscine, chelerythrine, Y-27632, and ML-7 hydrochloride is expressed as a percentage of the dexmedetomidine (10^−6^ M)-induced maximal contraction. The simultaneously measured values of dexmedetomidine-induced tension and [Ca^2+^]_i_ are expressed as the percentage of the 60 mM KCl-induced maximal contraction and [Ca^2+^]_i_, respectively. The contraction or [Ca^2+^]_i_ found in response to each concentration of dexmedetomidine was analyzed using repeated measures analysis of variance followed by Bonferroni’s post hoc test (Prism 5.0, GraphPad Software, San Diego, CA, USA). *p* Values less than 0.05 were considered statistically significant.

## 5. Conclusions

Taken together, these results suggest that the contraction induced by the α-2 adrenoceptor agonist dexmedetomidine involves calcium sensitization in isolated endothelium-denuded rat aortas, and this effect appears to be mediated by CPI-17 phosphorylation via Rho-kinase or PKC.

## Abbreviations

CPI-17	Phosphorylation-dependent inhibitory protein of myosin phosphatase
PKC	Protein kinase C
MLC_20_	20-kDa regulatory light chain of myosin
MLCP	Myosin light chain phosphatase
[Ca^2+^]_i_	Intracellular calcium concentration
VSMCs	Vascular smooth muscle cells
